# Analysis of predictors of adherent perinephric fat and its impact on perioperative outcomes in laparoscopic partial nephrectomy: a retrospective case–control study

**DOI:** 10.1186/s12957-021-02429-6

**Published:** 2021-11-04

**Authors:** Lu Fang, Huan Li, Tao Zhang, Rui Liu, Taotao Zhang, Liangkuan Bi, Dongdong Xie, Yi Wang, Dexin Yu

**Affiliations:** 1 Department of Urology, The Second Hospital of Anhui Medical University, 678 FuRong Road, Hefei, 230601 Anhui Province China; 2 Department of Radiology, The Second Hospital of Anhui Medical University, 678 FuRong Road, Hefei, 230601 Anhui Province China

**Keywords:** Adherent perinephric fat, Laparoscopic partial nephrectomy, Renal cell carcinoma, Mayo Adhesive Probability score

## Abstract

**Background:**

Adherent perinephric fat (APF), characterized by inflammatory fat surrounding the kidney, can limit the isolation of renal tumors and increase the operative difficulty in laparoscopic partial nephrectomy (LPN). The aim of this study was to investigate the predictors of APF and its impact on perioperative outcomes during LPN.

**Methods:**

A total of 215 consecutive patients undergoing LPN for renal cell carcinoma (RCC) from January 2017 to June 2019 at our institute were included. We divided these patients into two groups according to the presence of APF. Radiographic data were retrospectively collected from preoperative cross-sectional imaging. The perioperative clinical parameters were compared between the two groups. Univariate and multivariate analyses were performed to evaluate the predictive factors of APF.

**Results:**

APF was identified in 41 patients (19.1%) at the time of LPN. Univariate analysis demonstrated that APF was significantly correlated with the male gender (*P* = 0.001), higher body mass index (*P* = 0.002), lower preoperative estimated glomerular filtration rate (*P* = 0.004), greater posterior perinephric fat thickness (*P* **<** 0.001), greater perinephric stranding (*P* **<** 0.001), and higher Mayo Adhesive Probability (MAP) score (*P* **<** 0.001). The MAP score (*P* **<** 0.001) was the only variable that remained an independent predictor for APF in multivariate analysis. We found that patients with APF had longer operative times (*P* **<** 0.001), warm ischemia times (*P* = 0.001), and greater estimated blood loss (*P* = 0.003) than those without APF. However, there were no significant differences in surgical approach, transfusion rate, length of postoperative stay, complication rate, or surgical margin between the two groups.

**Conclusions:**

Several specific clinical and radiographic factors including the MAP score can predict APF. The presence of APF is associated with an increased operative time, warm ischemia time, and greater estimated blood loss but has no impact on other perioperative outcomes in LPN.

**Supplementary Information:**

The online version contains supplementary material available at 10.1186/s12957-021-02429-6.

## Background

According to the European Association of Urology (EAU) Renal Cancer Guidelines, partial nephrectomy (PN) is the preferred option for clinical stage T1 renal tumors (defined as tumors of ≤ 7 cm, confined to the renal parenchyma), when technically feasible [1]. With the increased availability and utilization of laparoscopic and robot-assisted techniques, minimally invasive PN (MIPN) has been identified as a safe and reproducible surgical approach, combining the advantages of decreased blood loss and hospital stay with similar oncological outcomes, compared with open PN [2–5]. In clinical practice, the treatment strategy of PN entails a complex decision process and is dependent on tumor and patient-specific factors [6, 7]. Several image-based tumor anatomical classification systems such as the PADUA classification system, the centrality index (C-index), and the RENAL nephrometry score system have been applied to evaluate the complexity and potential perioperative morbidity of PN [8–10]. Nevertheless, contemporary data assessing patient-specific factors that may also complicate the technical aspects of PN are limited.

Adherent perinephric fat (APF), a notable patient-specific factor, has attracted much attention over the years. APF, characterized by inflammatory fat surrounding the kidney, can restrict the isolation of renal tumors and increase the operative difficulty in PN [11–13]. Davidiuk et al. [14] proposed an image-based scoring algorithm called the Mayo Adhesive Probability (MAP) score to predict the presence of APF in robot-assisted PN (RAPN). However, the small cohort of patients enrolled and inadequate clinical predictors limit its extensive use. In this study, we sought to further investigate the predictive clinical and radiographic factors, including the MAP score, for APF, as well as to assess its impact on perioperative outcomes at the time of LPN.

## Methods

### Patient selection and data collection

With institutional review board approval, 300 consecutive patients who underwent LPN were prospectively analyzed in our institute from January 2017 to June 2019. The exclusion criteria were patients with an ipsilateral renal surgery history, who received preoperative neoadjuvant therapy, who had multifocal tumors, who had incomplete clinical information, and who had benign pathology. Eventually, 215 patients were enrolled in this study. Data were obtained regarding patients’ baseline clinical characteristics (gender, age, body mass index (BMI), hypertension, diabetes mellitus, tobacco use, dyslipidemia, preoperative serum creatinine, estimated glomerular filtration rate (eGFR)) and pathological characteristics (pathological stage, histological subtype, Fuhrman grade, renal capsular invasion, and perinephric fat invasion).

Radiographic data (tumor size, tumor location, RENAL nephrometry score, posterior perinephric fat thickness, perinephric fat stranding, and MAP score) were collected from preoperative CT imaging within 1 month before LPN by two authors (LH and WY) who were independently blinded to the results of the operative notes. Posterior perinephric fat thickness was measured at the level of the renal vein as the distance from the renal capsule to the posterior abdominal wall, following a previously described procedure [15]. Perinephric fat stranding was defined in accordance with a prior study [16] as a line area of soft tissue attenuation in the perinephric space and was graded according to severity. The final MAP score was generated from the sum of the two parameters described above with a range from 0 to 5 [14].

LPN procedures were carried out similar to previously published methods [17] by one senior experienced urologist surgeon (YDX) and divided briefly into the following three steps: step 1, establishing the laparoscopic approach and operating space; step 2, dissecting the perinephric fat to expose the tumor and renal hilar vessels; and step 3, resecting the tumor and closing the wound with hilar clamping.

**Fig. 1:**
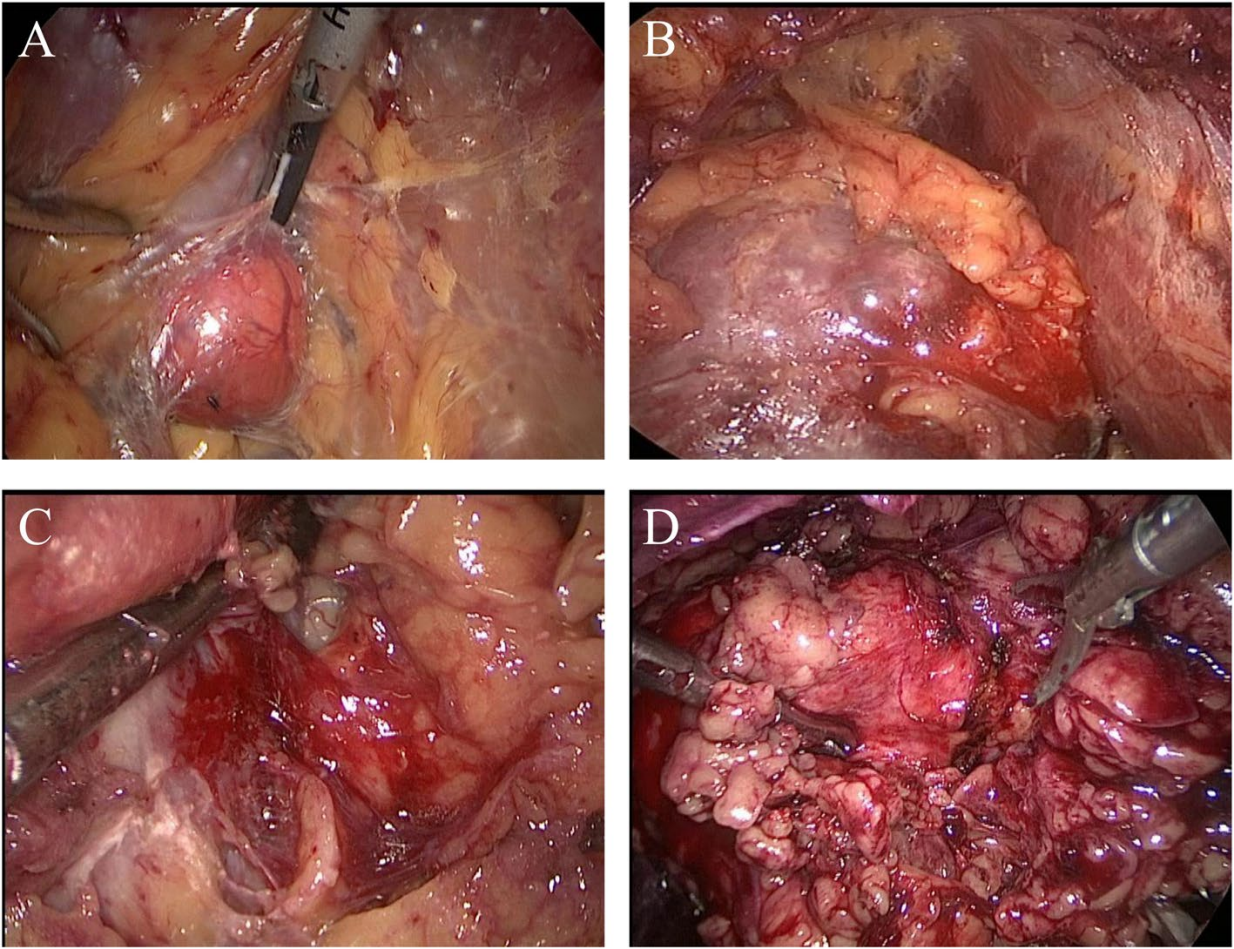
Grading of intraoperative adhesions of perinephric fat. **A** 0 points: no adhesions, blunt dissection with clear boundary and rare bleeding; **B** 1 point: mild adhesions, blunt dissection with clear boundary and mild bleeding; **C** 2 points: moderate adhesions, blunt and sharp dissection with still clear boundary, and moderate bleeding; **D** 3 points: severe adhesions, sharp dissection with blurred boundary, and obvious bleeding, even requiring subcapsular dissection

A scoring algorithm was made to describe intraoperative perinephric fat adhesion, shown in Fig. 1 (0 points: no adhesions, blunt dissection with clear boundary, and rare bleeding; 1 point: mild adhesions, blunt dissection with clear boundary, and mild bleeding; 2 points: moderate adhesions, blunt and sharp dissection with still clear boundary, and moderate bleeding; 3 points: severe adhesions, sharp dissection with blurred boundary, and obvious bleeding, even requiring subcapsular dissection). APF was defined by the surgeon intraoperatively as a score for 3 points.

The perioperative variables collected from medical records were surgical approach, operative time, warm ischemia time (WIT), estimated blood loss (EBL), transfusion, length of postoperative stay, postoperative complication, surgical margin, and the incidence rate of renal capsule rupture. Postoperative complications within 30 days of surgery were graded according to the Clavien-Dindo classification [18].

Follow-up was carried out by postoperative outpatient interview and telephone interview until September 2021, disease recurrence, death, or loss to follow-up. Overall survival (OS) was identified as the interval between surgery and last follow-up or death with any cause. Recurrence-free survival (RFS) was identified as the interval between surgery and last follow-up or disease recurrence.

### Statistical analysis

Normally and non-normally distributed continuous variables are summarized as means and standard deviations and medians (first quartile (Q1), third quartiles (Q3)), respectively. Categorical variables are reported as proportions with the number of patients. The distribution of continuous variables was checked by histograms and the Kolmogorov-Smirnov (K-S) test. Univariate and multivariate logistic regression analyses were performed to evaluate the predictive factors of APF. The differences in perioperative outcomes between patients with and without APF were compared using Student’s *t* test or the Mann–Whitney *U* test for continuous variables. The chi-squared test was used to compare categorical data. The survival curves were drawn according to the Kaplan-Meier method and compared using log-rank test. A *P* value < 0.05 indicates a statistically significant difference. All statistical analyses were performed using SPSS 20.0 statistical software (IBM, Chicago, IL, USA).

## Results

### Characteristics of patients

**Fig. 2:**
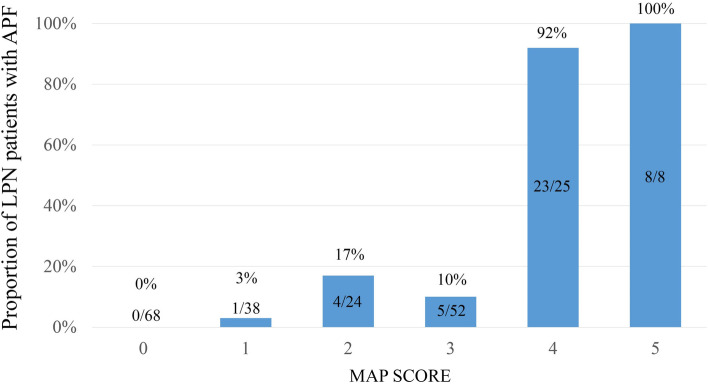
Proportion of laparoscopic partial nephrectomy (LPN) patients with adherent perinephric fat (APF) according to the MAP score

Of the 215 consecutive patients enrolled in the present study, 41 (19.1%) had APF identified during LPN. Table 1 highlights the baseline characteristics of the cohort in detail. The mean age at the time of diagnosis was 57 years, and the majority of patients were male (64.7%) and hypertensive (54.0%) and had a mean BMI of 24.1 kg/m^2^. The median preoperative serum creatinine was 70.0 μmol/l, and the mean eGFR was 117.7 ml/min/1.73 m^2^. The mean tumor size was 3.7 cm with a standard deviation of 1.5 cm. Pathological data revealed that most patients had a pT1 (91.1%) stage tumor and a clear cell RCC subtype (78.1%). The Fuhrman nuclear grade of RCC was marked on 174 patients, of whom 13 (7.5%) had grade I, 132 (75.9%) had grade II, and 29 (16.6%) had grade III. Fifty (23.3%) patients had renal capsular invasion, and 12 (5.6%) patients had perinephric fat invasion. Perinephric fat stranding was graded as none, mild/moderate, and severe in 51.2, 33.5, and 15.3% of patients, respectively. The mean posterior fat thickness was 1.1 cm, median nephrometry score was 6 (Q1, Q3: 6, 8) and median MAP score was 2 (Q1, Q3: 0, 3). The proportion of patients with APF for each level of the MAP score was as follows: 0 (*n* = 68), 0%; 1 (*n* = 38), 3%; 2 (*n* = 24), 17%; 3 (*n* = 52), 10%; 4 (*n* = 25), 92%; and 5 (*n* = 8), 100% (Fig. 2).

**Table 1:** Clinicopathological and radiographic characteristics stratified by the presence of adherent perinephric fat (APF)

Variable	Total (***N*** = 215)	APF group (***N*** = 41)	Non APF group (***N*** = 174)
Gender^a^			
Male	139 (64.7%)	36 (87.8%)	103 (59.2%)
Age (years)^b^	57.1 ± 13.4	59.9 ± 14.5	56.5 ± 13.1
BMI (kg/m^2^)^b^	24.1 ± 3.7	25.7 ± 3.7	23.7 ± 3.6
Hypertension^a^	116 (54.0%)	27 (65.9%)	89 (51.1%)
Diabetes mellitus^a^	52 (24.2%)	13 (31.7%)	39 (22.4%)
Tobacco use^a^	70 (32.6%)	17 (41.5%)	53 (30.5%)
Dyslipidemia^a^	88 (41.0%)	16 (39.0%)	72 (41.4%)
Preoperative creatinine (μmol/l)^c^	70.0 (58.0, 84.0)	80.0 (70.0, 97.5)	68.0 (54.0, 81.3)
Preoperative eGFR (ml/min/1.73 m^2^)^b^	117.7 ± 34.5	103.6 ± 30.1	121.1 ± 34.7
Tumor size (cm)^b^	3.7 ± 1.5	4.0 ± 1.4	3.6 ± 1.5
Tumor location^a^			
Left side	105 (48.8%)	22 (53.7%)	83 (47.7%)
Pathological stage^a^			
pT1a	131 (60.9%)	22 (53.7%)	109 (62.6%)
pT1b	65 (30.2%)	16 (39.0%)	49 (28.2%)
pT2a	6 (2.8%)	1 (2.4%)	5 (2.9%)
≥ pT3	13 (6.1%)	2 (4.9%)	11 (6.3%)
Histological subtype^a^			
Clear cell	168 (78.1%)	36 (87.8%)	132 (75.9%)
Papillary cell	6 (2.8%)	1 (2.4%)	5 (2.9%)
Chromophobe	12 (5.6%)	1 (2.4%)	11 (6.3%)
Other subtype	29 (13.5%)	3 (7.4%)	26 (14.9%)
^d^Fuhrman grade^a^			
I	13 (7.5%)	1 (2.7%)	12 (8.8%)
II	132 (75.9%)	27 (73.0%)	105 (76.6%)
III	29 (16.6%)	9 (24.3%)	20 (14.6%)
IV	0 (0.0%)	0 (0.0%)	0 (0.0%)
Renal capsular invasion^a^	50 (23.3%)	11 (26.8%)	39 (22.4%)
Perinephric fat invasion^a^	12 (5.6%)	2 (4.9%)	10 (5.7%)
RENAL nephrometry score^c^	6.0 (6.0, 8.0)	7.0 (6.0, 8.0)	6.0 (5.0, 8.0)
Posterior fat thickness (cm)^b^	1.1 ± 0.6	2.0 ± 0.6	0.9 ± 0.5
Perinephric stranding^a^			
None	110 (51.2%)	4 (9.8%)	106 (60.9%)
Mild/moderate	72 (33.5%)	13 (31.7%)	59 (33.9%)
Severe	33 (15.3%)	24 (58.5%)	9 (5.2%)
MAP score^c^	2.0 (0.0, 3.0)	4.0 (4.0, 4.0)	1.0 (0.0, 3.0)

*N*, number; *SD*, standard deviation; *Q*, quartile; *BMI*, body mass index; *eGFR*, estimated glomerular filtration rate; *MAP*, Mayo Adhesive Probability

^a^*N* (%)

^b^Mean ± SD

^c^Median (Q1, Q3)

^d^Fuhrman grade, 174/215 had recorded Fuhrman grade

### Predictors of APF

The clinical and radiographic variables predicting the presence of APF were evaluated by the logistic regression model. According to univariate analysis, APF significantly correlated with male gender (OR 4.963, *P* = 0.001), higher body mass index (OR 1.171, *P* = 0.002), lower preoperative estimated glomerular filtration rate (OR 0.983, *P* = 0.004), greater posterior perinephric fat thickness (OR 38.141, *P* **<** 0.001), greater perinephric stranding (OR 5.839; OR 70.667, *P* **<** 0.001), and higher MAP score (OR 8.945, *P* **<** 0.001) (Table 2). Based on these factors, multivariate analysis demonstrated that the MAP score (OR 8.870, *P* **<** 0.001) was the only variable that remained an independent predictor of APF (Table 3).

**Table 2:** Univariate logistic regression analysis for association of index variables and adherent perinephric fat (APF)

Variable	Univariate analysis	
	OR (95% CI)	***P*** value
Gender		**0.001**
Male	4.963 (1.857–13.264)	
Female	Reference	
Age (years)	1.020 (0.993–1.047)	0.145
BMI (kg/m^2^)	1.171 (1.058–1.296)	**0.002**
Hypertension		0.092
Yes	1.842 (0.905–3.749)	
No	Reference	
Diabetes mellitus		0.214
Yes	1.607 (0.761–3.396)	
No	Reference	
Tobacco use		0.178
Yes	1.617 (0.803–3.257)	
No	Reference	
Dyslipidemia		0.783
Yes	0.907 (0.452–1.819)	
No	Reference	
Preoperative creatinine (μmol/l)	1.010 (0.998–1.023)	0.101
Preoperative eGFR (ml/min/1.73 m^2^)	0.983 (0.972–0.995)	**0.004**
Tumor size (cm)	1.214 (0.970–1.521)	0.090
Tumor location		0.493
Left side	1.269 (0.642–2.511)	
Right side	Reference	
Pathological stage		0.290
pT1a	Reference	
> pT1a	1.448 (0.729–2.877)	
Histological subtype		0.103
ccRCC	Reference	
Non-ccRCC	0.437 (0.161–1.184)	
Fuhrman grade		0.234
I	Reference	
II	3.086 (0.384–24.783)	
III	5.400 (0.607–48.078)	
Renal capsular invasion		0.548
Yes	1.269 (0.583–2.761)	
No	Reference	
Perinephric fat invasion		0.828
Yes	0.841 (0.177–3.994)	
No	Reference	
RENAL nephrometry score	1.065 (0.885–1.282)	0.506
Posterior fat thickness (cm)	38.141 (12.524–116.156)	**< 0.001**
Perinephric stranding		**< 0.001**
None	Reference	
Mild/moderate	5.839 (1.821–18.719)	
Severe	70.667 (20.078–248.724)	
MAP score	8.945 (4.160–19.236)	**< 0.001**

*OR* odds ratio, *CI* confidence interval, *BMI* body mass index, *eGFR* estimated glomerular filtration rate, *ccRCC* clear cell renal cell carcinoma, *MAP* Mayo Adhesive Probability

**Table 3:** Multivariate logistic regression analysis of adherent perinephric fat (APF)

Variable	Multivariate analysis	
	OR (95% CI)	***P*** value
Gender (male vs female)	2.238 (0.611–8.200)	0.224
BMI (kg/m^2^)	0.957 (0.826–1.108)	0.555
Preoperative eGFR (ml/min/1.73 m^2^)	1.000 (0.983–1.016)	0.969
MAP score	8.870 (3.875–20.306)	**< 0.001**

*OR* odds ratio, *CI* confidence interval, *BMI* body mass index, *eGFR* estimated glomerular filtration rate, *MAP* Mayo Adhesive Probability

### Impact of APF on perioperative outcomes in LPN

As shown in Table 4, most of the patients received a retroperitoneal approach (82.8%) for LPN. Compared with the non-APF group, the APF group was associated with a significantly longer operative time (158.0 vs. 124.2 min, *P* **<** 0.001), warm ischemia time (17.9 vs. 13.5 min, *P* = 0.001), greater estimated blood loss (80 vs. 50 ml, *P* = 0.003), and higher incidence rate of renal capsule rupture (12.2% vs. 1.7%, *P* = 0.006). The rate of transfusion in this study population was relatively low (3.3%), and there was no difference in the length of postoperative stay. Overall, 30-day complications and positive surgical margins occurred in 31.6 and 2.3% of patients, respectively, with no difference between the two groups.

**Table 4:** Impact of adherent perinephric fat (APF) on perioperative outcomes in laparoscopic partial nephrectomy (LPN)

Variable	Total (***N*** = 215)	APF group (***N*** = 41)	Non APF group (***N*** = 174)	***P*** value
Surgical approach^a^				0.344
Retroperitoneal	178 (82.8%)	36 (87.8%)	142 (81.6%)	
Transperitoneal	37 (17.2%)	5 (12.2%)	32 (18.4%)	
Operative time (min)^b^	130.7 ± 41.0	158.0 ± 38.3	124.2 ± 39.0	**< 0.001**
Warm ischemia time (min)^b^	14.3 ± 7.3	17.9 ± 7.2	13.5 ± 7.2	**0.001**
Estimated blood loss (ml)^c^	50.0 (30.0, 100.0)	80.0 (50.0, 150.0)	50.0 (30.0, 80.0)	**0.003**
Transfusion^a^	7 (3.3%)	3 (7.3%)	4 (2.3%)	0.254
Length of postoperative stay (days)^c^	8.0 (7.0, 9.0)	8.0 (7.0, 9.0)	8.0 (7.0, 9.0)	0.191
Postoperative complication^a^	68 (31.6%)	12 (29.2%)	56 (32.2%)	0.746
Clavien-Dindo I–II	64 (29.8%)	11 (26.8%)	53 (30.5%)	
Clavien-Dindo III–IV	4 (1.8%)	1 (2.4%)	3 (1.7%)	
Surgical margin^a^				0.957
Positive	5 (2.3%)	1 (2.4%)	4 (2.3%)	
Negative	210 (97.7%)	40 (97.6%)	170 (97.7%)	
Renal capsule rupture^a^	8 (3.7%)	5 (12.2%)	3 (1.7%)	**0.006**

^a^*N* (%)

^b^Mean ± SD

^c^Median (Q1, Q3)

### Association between APF and oncological outcomes of RCC patients

**Fig. 3:**
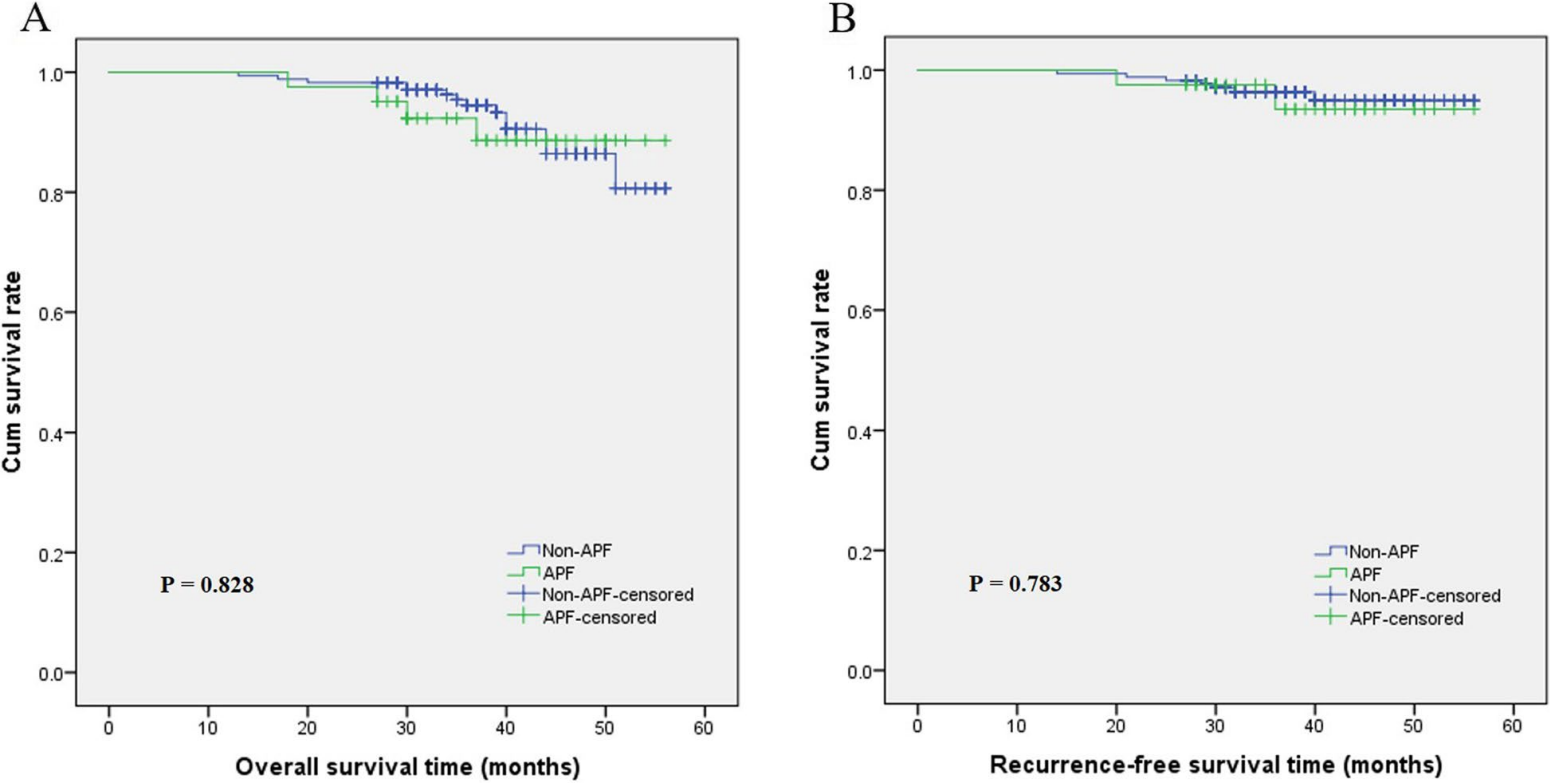
Kaplan-Meier method was applied to analyze the overall survival (OS) and recurrence-free survival (RFS). There was no significant difference between the APF group and non-APF group in OS (*P* = 0.828) and RFS (*P* = 0.783), respectively

The association between intraoperative APF and the prognosis of RCC patients were analyzed by the Kaplan-Meier method. The mean and median follow-up times were 38.5 and 37.0 months. Figure 3 shows the survival curves for OS and RFS and suggested that there was no significant difference between the APF group and non-APF group in OS (*P* = 0.828) and RFS (*P* = 0.783), respectively.

## Discussion

Since LPN was first reported by Winfield et al. in 1993 [19], it has increasingly become a preferred approach for the surgical management of cT1 renal masses, given evidence supporting similar oncologic efficacy and better perioperative outcomes compared with open PN [2–5]. However, LPN is technically challenging because it requires not only a negative surgical margin resection but time-dependent renal reconstruction [20–23]. The implementation of LPN is affected by a variety of factors, including tumor size, location, depth, and its relationship to renal hilar vessels and the urinary collecting system. Several scoring systems that quantify renal tumor anatomical factors have been developed to evaluate the surgical complexity and perioperative outcomes. Among them, the PADUA classification system, C-index, and RENAL nephrometry score system are the most widely used algorithms [8–10]. Nevertheless, these algorithms focus entirely on tumor-specific factors and ignore patient-specific factors that may also play an essential role in the LPN procedure.

It is not an uncommon occurrence when performing PN that thick and adherent perinephric adipose tissues within the Gerota’s fascia complicate the exposure of the renal parenchyma and tumor. As a notable patient-specific factor, APF has attracted much attention in the last decade. However, the definition of APF is still lack of a uniform standard. A series of definitions have been reported in the literature, such as inflammatory perirenal fat adhering to the renal parenchyma that makes kidney dissection difficult and results in bleeding and decapsulation [13] and perirenal fat within the Gerota’s fascia requiring subcapsular dissection [14]. Differing from these relatively subjective definitions of APF, we made a scoring index based on the macroscopic appearance to describe intraoperative adhesions of perinephric fat, which may help to universalize its definition.

Prior studies have demonstrated that the presence of APF can result in adverse perioperative outcomes during MIPN. Kocher et al. revealed a statistically significant association among APF, longer operative time, and higher estimated blood loss [12]. Additionally, Khene et al. emphasized an elevated risk of conversion to open surgery or radical nephrectomy in patients with APF [13]. Similarly, in a large cohort of patients with RCC that underwent LPN, our data also identified APF as significantly correlated with an increased estimated blood loss (*P* = 0.003) and operative time (*P* **<** 0.001). We observed that APF had no impact on the surgical margins and postoperative complications. Additionally, under comparable surgeons’ experience and tumor complexity, the warm ischemia time in cases with APF was 4 min longer than in those without APF (*P* = 0.001), which agreed with the finding from Borregales et al. [24]. The possible explanation for these results is as follows; adherent perinephric adipose tissues are more brittle and prone to bleeding, and when exposing and resecting the renal tumor, a blurred boundary caused by APF usually requires sharp dissection and an expanded scope of resection to ensure a negative surgical margin (Fig. 1), which further increases bleeding and suture difficulty and prolongs the warm ischemia time and operative time.

In view of the adverse perioperative outcomes associated with APF, a series of studies have been performed to investigate its physiologic mechanism and predictive factors. While the underlying pathogenesis of APF remains unclear, studies suggest that inflammation, idiopathic fibrosis, and the autoimmune response may account for APF [25]. Previous basic research has indicated the contributions of inflammation and fibrosis to abnormal adipose tissue expansion in obesity. Inflammation can lead to hypoxia and fibrosis in adipocytes, which can, in turn, promote the migration of immune cells into adipose depots [26]. As an index of obesity, the role of BMI in predicting APF is contentious. According to our univariate analysis, BMI was found to be closely associated with APF (*P* = 0.002), and similar findings were confirmed in other studies [13, 14]. However, it has also been argued that there is no significant correlation between BMI and APF [12], probably because BMI does not accurately reflect the variation in fat distribution, especially visceral fat (obesity), which is strongly related to metabolic syndrome [27]. This variation manifests in gender as well, as women have more subcutaneous fat than men, while men have more perirenal fat than women [15]. As a result, most studies, including ours, indicate that males have a higher incidence of APF (*P* = 0.001). Furthermore, other clinical factors predicting the presence of APF, such as age, cardiovascular disease, and diabetes mellitus, have been reported in a few studies [12–14, 24]. Notably, in the present study, we found that APF correlated with a decreased preoperative level of eGFR (*P* = 0.004), which may suggest that a chronic inflammatory response participates in the formation of APF [28].

To further investigate the predictors of APF, the radiographic parameters were analyzed at the same time. Posterior perinephric fat thickness, as a measurement of intra-abdominal fat, has a significant relationship with APF and complications of MIPN [11, 14]. Perinephric fat stranding was initially observed in cross-sectional imaging under inflammatory conditions, such as pyelonephritis and ureteral obstruction [16], and has also been identified in cases of APF recently. Based on these two radiographic factors, a semiquantitative scoring system called the MAP score has been proposed to predict APF during RAPN [14]. Our multivariate analysis revealed that the MAP score was an independent predictor of APF (*P* **<** 0.001), providing concomitant external validation in a large cohort of LPN.

As mentioned above, the pathogenesis of APF may correlate with inflammation, while cancer-related inflammation is known to be involved in tumor development and progression, including RCC [29]. Kocher et al. showed that APF was associated with malignant renal histology (versus benign disease) [12], and Thiel et al. revealed that high MAP scores were related to decreased progression-free survival of RCC [30]. Interestingly, our study failed to elucidate the association between APF and tumor-aggressive behaviors, and the oncological outcomes.

There are several limitations in this study. First, considering the difference of treatment strategy between benign and malignant renal tumors, we excluded benign tumors in the study. Second, the limited number of single-center patients and the relatively strong correlations among previously mentioned clinical factors made the application of multivariate model analysis challenging. Third, our definition of APF may require further validation with multicenter and larger cohort studies.

## Conclusions

APF can be preoperatively predicted with the comprehensive assessment of several specific clinical and radiographic factors, including male gender, higher BMI, and the MAP score. The presence of APF is associated with an increased operative time, warm ischemia time, and greater estimated blood loss but has no impact on other perioperative outcomes in LPN. Consequently, the accurate evaluation and adequate understanding of APF will be helpful to counsel patient selection and improve outcomes.

## Supplementary Information

**Additional file 1 : Table S1.** Characteristics of current literatures on the study of adherent perinephric fat in partial nephrectomy

**Additional file 2 : Table S2.** Impact of adherent perinephric fat on perioperative outcomes in laparoscopic partial nephrectomy after homogeneous adjustment for BMI

## Data Availability

The datasets generated and analyzed during the current study are not publicly available because we are conducting further investigations but are available from the corresponding author on reasonable request.

## Abbreviations

APF: Adherent perinephric fat
LPN: Laparoscopic partial nephrectomy
RCC: Renal cell carcinoma
MAP: Mayo Adhesive Probability
CT: Computed tomography
BMI: Body mass index
eGFR: Estimated glomerular filtration rate
WIT: Warm ischemia time
EBL: Estimated blood loss
