# Supplementary material for: Analysis of predictors of adherent perinephric fat and its impact on perioperative outcomes in laparoscopic partial nephrectomy: a retrospective case–control study
Source: World J Surg Oncol. 2021 Nov 4;19:319. doi: 10.1186/s12957-021-02429-6 (PMC8567560; doi:10.1186/s12957-021-02429-6)
Supplement: Supplementary file 1 — Additional file 1 : Table S1. Characteristics of current literatures on the study of adherent perinephric fat in partial nephrectomy [file 12957_2021_2429_MOESM1_ESM.docx]

Table S1. Characteristics of current literatures on the study of adherent perinephric fat in partial nephrectomy.

| **Study/No.** | **Author** | **Publish**  **time** | **Country** | **Study duration** | **Study**  **design** | **Study range** | **Surgical approach** | **Tumor type** | **Total No.** | **No. of APF** | **Definition of APF** |
| --- | --- | --- | --- | --- | --- | --- | --- | --- | --- | --- | --- |
| 1 | Bylund  et al. | 2013 | USA | 2005-2011 | Retrospective | Single- center | OPN and MIPN and laparoscopic cryoablation | RCC and benign renal tumor | 29 | 16 | the perinephric fat was described as ‘‘dense,’’ ‘‘adherent,’’ ‘‘sticky’’ or similar terms. |
| 2 | Davidiuk et al. | 2014 | USA | 2008-2014 | Prospective | Single- center | RAPN | RCC and benign renal tumor | 100 | 30 | perirenal fat within the Gerota fascia requiring subcapsular dissection for exposure of the renal parenchyma and renal tumor. |
| 3 | Khene  et al. | 2015 | France | 2010-2014 | Retrospective | Single- center | RAPN | Renal tumor | 202 | 80 | inflammatory perirenal fat adhering to the renal parenchyma that makes kidney dissection difficult and results in bleeding and decapsulation. |
| 4 | Kocher  et al. | 2016 | USA | 2000-2014 | Retrospective | Single- center | RAPN and LPN | RCC and benign renal tumor | 245 | 26 | “adherent” or “sticky” perinephric fat within Gerota’s fascia. |
| 5 | Martin  et al. | 2017 | France | 2009-2015 | Retrospective | Single- center | OPN | Renal tumor | 86 | 43 | difficulties with dissecting APF. |
| 6 | Dariane  et at. | 2017 | France | 2014-2015 | Prospective | Single- center | RAPN and OPN | Clear cell RCC and  Non-clear cell tumor | 125 | 51 | 0-to-3 dedicated scale (APF defined by a score ≥2) (score 0: no perinephric fat; score 1: non-APF; score 2: APF with no decapsulation of the kidney; score 3: sticky APF responsible for decapsulation during dissection). |
| 7 | Kawamura  et al. | 2018 | Japan | 2011-2013 | Retrospective | Single- center | gasless laparo-endoscopic single-port clampless sutureless PN | RCC and benign renal tumor | 231 | 40 | perirenal fat within the Gerota fascia that was adhering to the renal parenchyma and difficult to dissect for exposure of the renal tumor. |
| 8 | Borregales  et al. | 2019 | USA | 2009-2014 | Retrospective | Two-  center | RAPN, LPN and OPN | RCC and benign renal tumor | 495 | 95 | the presence of “dense,” “adherent,” or “sticky” perinephric fat noted by the surgeon at the time of dissection. |
| 9 | Haehn  et al. | 2021 | USA | 2009-  2020 | Retrospective | Single- center | OPN | RCC and benign renal tumor | 87 | 43 | the requirement of subcapsular dissection for full exposure of the renal tumor. |

APF, adherent perinephric fat; OPN, open partial nephrectomy; MIPN, minimally invasive partial nephrectomy; RAPN, robot-assisted partial nephrectomy (RAPN); LPN, laparoscopic partial nephrectomy; RCC, renal cell carcinoma.

**References**

1. Bylund JR, Qiong H, Crispen PL, Venkatesh R, Strup SE: **Association of clinical and radiographic features with perinephric "sticky" fat.** *J Endourol* 2013, **27:**370-373.

2. Davidiuk AJ, Parker AS, Thomas CS, Leibovich BC, Castle EP, Heckman MG, Custer K, Thiel DD: **Mayo adhesive probability score: an accurate image-based scoring system to predict adherent perinephric fat in partial nephrectomy.** *Eur Urol* 2014, **66:**1165-1171.

3. Khene ZE, Peyronnet B, Mathieu R, Fardoun T, Verhoest G, Bensalah K: **Analysis of the impact of adherent perirenal fat on peri-operative outcomes of robotic partial nephrectomy.** *World J Urol* 2015, **33:**1801-1806.

4. Kocher NJ, Kunchala S, Reynolds C, Lehman E, Nie S, Raman JD: **Adherent perinephric fat at minimally invasive partial nephrectomy is associated with adverse peri-operative outcomes and malignant renal histology.** *BJU Int* 2016, **117:**636-641.

5. Martin L, Rouviere O, Bezza R, Bailleux J, Abbas F, Schott-Pethelaz AM, Ruffion A, Paparel P: **Mayo Adhesive Probability Score Is an Independent Computed Tomography Scan Predictor of Adherent Perinephric Fat in Open Partial Nephrectomy.** *Urology* 2017, **103:**124-128.

6. Dariane C, Le Guilchet T, Hurel S, Audenet F, Beaugerie A, Badoual C, Tordjman J, Clément K, Urien S, Pietak M, et al: **Prospective assessment and histological analysis of adherent perinephric fat in partial nephrectomies.** *Urol Oncol* 2017, **35:**39.e39-39.e17.

7. Kawamura N, Saito K, Inoue M, Ito M, Kijima T, Yoshida S, Yokoyama M, Ishioka J, Matsuoka Y, Kihara K, Fujii Y: **Adherent Perinephric Fat in Asian Patients: Predictors and Impact on Perioperative Outcomes of Partial Nephrectomy.** *Urol Int* 2018, **101:**437-442.

8. Borregales LD, Adibi M, Thomas AZ, Reis RB, Chery LJ, Devine CE, Wang X, Potretzke AM, Potretzke T, Figenshau RS, et al: **Predicting Adherent Perinephric Fat Using Preoperative Clinical and Radiological Factors in Patients Undergoing Partial Nephrectomy.** *Eur Urol Focus* 2019.

9. Haehn DA, Bajalia EM, Cockerill KJ, Kahn AE, Ball CT, Thiel DD: **Validation of the Mayo Adhesive Probability score as a predictor of adherent perinephric fat and outcomes in open partial nephrectomy.** *Transl Androl Urol* 2021, **10:**227-235.
