# Supplementary material for: Analysis of predictors of adherent perinephric fat and its impact on perioperative outcomes in laparoscopic partial nephrectomy: a retrospective case–control study
Source: World J Surg Oncol. 2021 Nov 4;19:319. doi: 10.1186/s12957-021-02429-6 (PMC8567560; doi:10.1186/s12957-021-02429-6)
Supplement: Supplementary file 2 — Additional file 2 : Table S2. Impact of adherent perinephric fat on perioperative outcomes in laparoscopic partial nephrectomy after homogeneous adjustment for BMI [file 12957_2021_2429_MOESM2_ESM.docx]

Table S2. Impact of adherent perinephric fat on perioperative outcomes in laparoscopic partial nephrectomy after homogeneous adjustment for BMI.

| **Variable** | **APF group**  **(N = 38)** | **Non APF group**  **(N = 76)** | **P value** |
| --- | --- | --- | --- |
| BMI (kg/m^2^)^+^ | 25.5 ± 3.0 | 25.4 ± 2.7 | **0.849** |
| Surgical approach^*^ |  |  | 0.586 |
| Retroperitoneal | 33 (86.8%) | 63 (82.9%) |  |
| Transperitoneal | 5 (13.2%) | 13 (17.1%) |  |
| Operative time (min)^+^ | 156.1 ± 38.6 | 128.4 ± 36.8 | **< 0.001** |
| Warm ischemia time (min)^+^ | 18.4 ± 7.2 | 13.3 ± 7.7 | **0.001** |
| Estimated blood loss (ml)^#^ | 90.0 (50.0, 150.0) | 50.0 (40.0, 80.0) | **0.010** |
| Transfusion^*^ | 3 (7.9%) | 2 (2.6%) | 0.419 |
| Length of postoperative stay (days)^#^ | 8.0 (7.0, 9.0) | 8.0 (7.0, 9.0) | 0.443 |
| Postoperative complication^*^ | 12 (31.6%) | 23 (30.3%) | 0.907 |
| Clavien-Dindo I-II | 11 (28.9%) | 20 (26.3%) |  |
| Clavien-Dindo III-IV | 1 (2.7%) | 3 (4.0%) |  |
| Surgical margin^*^ |  |  | 1.000 |
| Positive | 1 (2.6%) | 1 (1.3%) |  |
| Negative | 37 (97.4%) | 75 (98.7%) |  |

^*^N (%); ^+^Mean ± SD; ^#^Median (Q1, Q3).
